# TESTLoc: protein subcellular localization prediction from EST data

**DOI:** 10.1186/1471-2105-11-563

**Published:** 2010-11-15

**Authors:** Yao-Qing Shen, Gertraud Burger

**Affiliations:** 1 Robert-Cedergren Center for Bioinformatics and Genomics; Biochemistry Department, Université de Montréal, 2900 Edouard-Montpetit, Montreal, QC, H3T 1J4, Canada

## Abstract

**Background:**

The eukaryotic cell has an intricate architecture with compartments and substructures dedicated to particular biological processes. Knowing the subcellular location of proteins not only indicates how bio-processes are organized in different cellular compartments, but also contributes to unravelling the function of individual proteins. Computational localization prediction is possible based on sequence information alone, and has been successfully applied to proteins from virtually all subcellular compartments and all domains of life. However, we realized that current prediction tools do not perform well on partial protein sequences such as those inferred from Expressed Sequence Tag (EST) data, limiting the exploitation of the large and taxonomically most comprehensive body of sequence information from eukaryotes.

**Results:**

We developed a new predictor, TESTLoc, suited for subcellular localization prediction of proteins based on their partial sequence conceptually translated from ESTs (EST-peptides). Support Vector Machine (SVM) is used as computational method and EST-peptides are represented by different features such as amino acid composition and physicochemical properties. When TESTLoc was applied to the most challenging test case (plant data), it yielded high accuracy (~85%).

**Conclusions:**

TESTLoc is a localization prediction tool tailored for EST data. It provides a variety of models for the users to choose from, and is available for download at http://megasun.bch.umontreal.ca/~shenyq/TESTLoc/TESTLoc.html

## Background

In eukaryotes, the majority of proteins are encoded in the nuclear genome and translated on ribosomes in the cytosol. Proteins are then transported to different subcellular locations, such as the nucleus, mitochondria, chloroplasts, peroxisomes, etc., where they perform their particular roles in various biological processes. Knowledge of subcellular location is an important asset in the annotation of newly discovered proteins, as it bears clues about a protein's function. Further, knowing the location of proteins and their molecular function allows us to infer where in the cell the corresponding biological process takes place, what the physiological role of this process may be, and how the various processes are spatially integrated. Finally, information on the makeup of proteomes from bacteria-derived organelles (mitochondria and chloroplasts) helps to elucidate the migration of protein-coding genes from the endosymbiont to the host.

A variety of experimental approaches are available today for identifying the subcellular localization of proteins, for example, co-expression of fluorescent proteins [1,2], immunofluorescence labeling [3], gene knockout/knockdown [4], and proteomics techniques such as liquid-chromatography-tandem mass spectrometry (LC-MS/MS) [5,6]. However, for most species, large-scale experimental identification of protein subcellular localization remains too expensive or unfeasible. This has set the stage for bioinformatics approaches to predict localization *in silico*.

Can localization of a protein be confidently inferred via finding a homolog of known location by BLAST [7]? A previous study indicated that localization can be predicted with up to 90% accuracy when BLAST identity is 50% or more, but that it falls short for more distant sequences (e.g., only 50% accuracy for 20% local identity, Additional file 1) [8]. Further, this approach ignores established biological knowledge that homologous proteins are not necessarily located in the same cellular compartment. For example, homologous beta oxidation enzymes are targeted to mitochondria in human and peroxisomes in yeast [9]. Most importantly, the BLAST approach fails for divergent and novel proteins as they do not find significant matches in databases (see Additional file 1). For all these reasons, the bioinformatics community turned to more suited approaches for protein localization prediction.

Today, more than 20 dedicated tools are available for *in silico *protein localization prediction based on annotation or solely the sequence of proteins (Additional file 2). Annotation information includes textual description taken from the SWISSPROT database, the Gene Ontology database, or PubMed literature [10-12]. Also used for localization prediction is co-occurrence of functional motifs or structural domains in proteins [13,14]. Sequence-based tools recognize specific targeting signals that guide proteins to different cellular compartments [15-19]. Alternatively, proteins are classified according to single amino acid frequency [20,21], dipeptide and gapped amino acid pair composition [22-25], or physicochemical properties of amino acids [26]. More recently published predictors combine different protein features [27-31], or integrate annotation with sequences-based prediction [32,33]. Finally, meta-predictors combine predictions from several heterogeneous tools [34-36].

Two recent studies evaluated the performance of available localization predictors using datasets that contain only sequences not included in, nor similar to, those in the training sets of these predictors [37,38]. One identified as best performing tools BaCelLo [39], LOCtree [29], Protein Prowler [18], TargetP [16], and Wolf-PSORT [40], and the other evaluated BaCelLo, YLoc [38], MuitiLoc2 [32], and KnowPred [41] as best (for sequence features and computational methods used, see Additional file 2). In general, these tools have lower performance on data from plants compared to non-photosynthetic organisms such as animals and fungi, and this is due to the presence of mitochondria plus chloroplasts in the cell of plants. Both organelles descend from endosymbiotic bacteria and have their own machineries for protein import, DNA replication, and gene expression. This makes it difficult for the tools to distinguish the proteins from the two organelles.

*In silico *localization prediction tools use full-length protein sequences that are usually inferred from genome sequence. Yet, for many eukaryotic groups of interest are only EST (Expressed Sequence Tag) data available, and it is unlikely that their genomes will be sequenced soon [42]. (For relevant public databases see dbEST of NCBI [43], The Gene Index Project (TGI) database [44], and the Taxonomically Broad EST DataBase (TBestDB) [45]). When attempting to use available localization prediction tools on protein sequences conceptually translated from ESTs, we realized that prediction accuracy is generally very low. We tested the performance of seven state-of-the-art tools with proteins inferred from plant ESTs, and the overall accuracies were below 50% (Table 1). This is not surprising, because these tools have been designed for full-length proteins and not for ESTs, which often represent only partial coding regions with an average length of ~200 residues. Further, EST-inferred proteins (referred to as EST-peptides from here on) may have an amino acid composition that differs from that of the corresponding full-length proteins. More importantly, EST-peptides often lack the N-terminal region of the corresponding proteins, which usually contains the targeting signal.

**Table 1:** Performance of available tools and TESTLoc on plant EST-peptides^1^

Predictors	chl^2^		cyt		end		ext		mit		nuc		per		pla		vac	
	
	SN	PPV	SN	PPV	SN	PPV	SN	PPV	SN	PPV	SN	PPV	SN	PPV	SN	PPV	SN	PPV
TargetP	19	62					29	25	18	44								
Protein Prowler	14	66					34	32	26	60			25	100				
BaCelLo	61	69	50	28			60	32	9	90	88	39						
Wolf-PSORT	27	41	60	27	0	0	18	36	8	58	65	71	16	50	0	0	0	0
YLoc	25	77	84	13	22	13	52	98	35	84	82	80	50	26	0	0	15	31
KnowPred	NA	NA	71	23	0	0	23	36	61	49	86	40	58	54	0	0	NA	NA
MultiLoc2	14	81	84	12	9	8	44	48	18	37	48	67	67	10	0	0	45	54
BLAST^3^	76	97	77	62	64	100	81	95	57	87	77	96	25	60	0	0	0	0
**TESTLoc**	**99**	**99**	**50**	**88**	**20**	**20**	**71**	**98**	**86**	**71**	**75**	**96**	**50**	**21**	**30**	**5**	**63**	**100**

^1 ^Numbers in %. Bold numbers are the result of the here described TESTLoc, which is tailored for ESTs. The results of full-length proteins are compiled in Additional file 8

^2 ^Abbreviations: chl, chloroplast; cyt, cytosol; end, endoplasmatic reticulum; ext, extracellular space; mit, mitochondrion; nuc, nucleus; per, peroxisome; pla, plasma membrane; vac, vacuole; SN, sensitivity; PPV, positive predictive value

^3 ^Note that all test data have homologs in databases, which in practice is rarely the case; see text

Finally BLAST, which we showed above to be unsuited for localization prediction of full-length proteins, is equally unsuited on EST-peptide data. Even at sequence identity levels above 90%, the class-averaged accuracy for plant ESTs was below 75% (Additional file 1). In practice, the accuracy would be even lower as EST projects often discover novel proteins that lack matches in databases. For example, in a large-scale protist EST project, more than 60% of ESTs could not find informative matches [45,46]. For the ESTs from such projects, the overall accuracy of localization prediction by BLAST would thus be less than 30%.

We set out to develop a method that is tailored for predicting subcellular localization based on ESTs. As a test case we used plant data, which, as mentioned above, are more challenging than those from non-photosynthetic taxa. The methodology we developed can be readily applied to ESTs from any taxonomic groups, and the models we constructed can be easily retrained with sequences from a particular taxon of interest.

## Methods

### Datasets

We used two datasets: data from all plants to build and evaluate the localization prediction models (input data: EST-peptides), and *Arabidopsis*-only data to evaluate the combined prediction of ORFs and localization (input data: EST nucleotide sequences).

#### Collection of protein sequences of experimentally verified subcellular location

From SWISSPROT, we collected full-length *Arabidopsis *proteins localized in nine subcellular compartments: chloroplasts (chl), cytosol (cyt), endoplasmic reticulum (end), extracellular space (ext), mitochondria (mit), nucleus (nuc), peroxisomes (per), plasma membrane (pla), and vacuole (vac). Protein sequences were selected by the following criteria: 1) they are encoded by the nuclear genome; 2) their subcellular localization is experimentally verified; and 3) the localization annotation is unambiguous (i.e., terms like "probable" or "possible" are absent from their subcellular localization annotation). These are strict criteria in order to avoid false positives in the dataset. No bias was detected as to the functional categories of proteins collected this way compared to other studies [47] (Additional file 3).

#### *Arabidopsis* ESTs dataset

The ESTs corresponding to the above *Arabidopsis *proteins were found via a similarity search by BLASTX in dbEST of GenBank, using a procedure illustrated in Additional file 4. When the aligned region of an EST was longer than 90% of its total length and the amino acid identity between the protein and the translated EST was >90%, we regarded the pair of EST and protein as belonging to the same gene. The selected ESTs were translated by EMBOSS Transeq [48] into amino acid sequences in the frame indicated by the BLASTX alignment. Sequence redundancy within the collected data was reduced by the tool CD-hit [49] so that no pair of sequences shares more than 60% identity. We obtained a dataset of 386 ESTs. Table 2 compiles the number of instances in each subcellular class.

**Table 2:** Number of EST-peptides used in this study

dataset	chl	cyt	end	ext	mit	nuc	per	pla	vac	total
*Arabidopsis*	97	53	5	9	167	41	5	4	5	386
Expanded plant data	679	122	11	48	309	260	12	7	29	1477

#### Plant ESTs dataset

Using the same procedure as described above for *Arabidopsis*, we generated EST-peptides for all other plants with known localization (440 sequences). These combined with the above described *Arabidopsis *dataset constitute the plant dataset (826 sequences), which was then used to build the expanded plant dataset as specified in the following.

#### Expanded plant ESTs dataset

Machine learning schemes perform better when trained with larger datasets. But in practice, the size of training data is often limited by their availability. We noticed that in our initial collection of EST-peptides, a number of proteins with known subcellular location were absent, because they have no corresponding EST sequences in public databases. To construct a training set with optimal coverage, the missing EST-peptides were substituted by artificial ones, generated by breaking up full-length proteins into overlapping pieces of ~200 residues. In this way, we almost tripled the size of training data. The procedure is described below.

**Figure 1:**
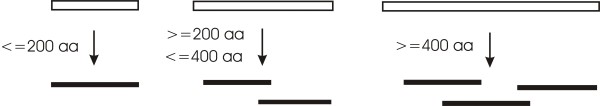
**Fragmentation procedure of plant protein sequences in order to expand the EST-peptide dataset**. Open bars, full-length proteins; filled bars, fragmented protein sequences. Proteins shorter than 200 residues remained unchanged. Proteins ranging from 200 to 400 residues were fragmented into two pieces. Proteins longer than 400 residues were fragmented into three pieces. See text for details.

The collected full-length protein sequences from plants were processed according to the following rules (Figure 1):

(1) When a sequence was shorter than 200 residues, it remained unchanged.

(2) When a sequence was 200 to 400 residues long, fragments of length ranging from 140 to 260 residues were generated from both the N-terminus and C-terminus. The range was based on a survey of the length distribution of ESTs, which revealed a mean of ~600 nt with a standard deviation of ~180 nt. The N-terminal fragment started within 80 residues from the first methionine, and the C-terminal fragment ended at the last amino acid. This simulated the nature of ESTs, which usually contain the complete C-terminal, but lack the N-terminal region.

(3) When a sequence was longer than 400 residues, an additional central fragment was generated starting anywhere downstream of the first 80 residues, but before the middle position of the original sequence.

The fragmented protein sequences were combined with EST-derived peptides and clustered by CD-hit (Li and Godzik, 2006) using a threshold of 60%. The final dataset contains sequences from 41 species.

### Localization prediction by dedicated tools and BLAST

We collected the best-performing localization prediction tools: TargetP, Protein Prowler, BaCelLo, Wolf-PSORT, YLoc, KnowPred, and MultiLoc2 (see Introduction), and tested their performance on the expanded plant dataset. The results of Wolf-POSRT and MultiLoc2 were obtained from a locally installed version. The prediction of YLoc was provided by its author. For the remaining tools, the results were obtained from their corresponding web-server.

To assess how BLAST performs for localization prediction of ESTs, the EST data were blasted against proteins in SWISSPROT, and the localization information of the second match (the first match is the same protein as the query ESTs, see data collection above) is transferred to the ESTs.

### Implementation of Support Vector Machine

#### Features used to represent the peptide sequences for input of SVM

##### Physicochemical properties

Physicochemical properties of amino acids in a protein can be represented by amino acid indices (AAindex, developed by the Amino Acid Index Database (http://www.genome.jp/dbget-bin/show_man?aaindex)). The database currently contains 494 features for each amino acid (such as values of hydrophobicity, bulkiness, alpha-helix, turn, beta-sheet propensity, etc.). For each amino acid feature, its value was added up for the whole sequence, and was normalized by the sequence length. Subsequently, each EST-peptide was converted into a 494-dimension vector.

##### Amino acid composition

Six different types of amino acid composition were calculated. These include the frequency of individual amino acids (1^st ^order), di-peptides (2^nd ^order), tri-peptides (3^rd ^order), tetra-peptides (4^th ^order), penta-peptides (5^th ^order), and hexa-peptides (6^th ^order) in the input sequence.

##### Grouped amino acid composition

Amino acids were grouped according to their properties (Table 3). The alphabet of 20 amino acids was replaced by an alphabet of size eight (group C) or size ten (group D). Group C classified amino acids according to their chemical properties, which have shown good performance for localization prediction of full-length proteins [50]. Group D classified amino acids according to their structure [51]. After converting EST sequences using these new alphabets, the composition of amino acid groups was calculated from 1^st ^to 8^th ^order.

**Table 3:** Amino acids grouped according to their chemical properties

Group C, chemical properties		Group D, Devlin structural properties		
**Property**	**Amino acid**	**Superstructure**	**Structure**	**Amino acid**

Acidic	D, E	Monoamino Moncarboxylic		G, A
Basic	H, K, R		Unsubstituted	V, L, I
Aromatic	F,W, Y		Heterocyclic	P, F
Small hydroxyl	S, T		Aromatic	W, Y
Sulphur containing	C, M		Thioether	M
Aliphatic1	A, G, P		Hydroxy	S, T
Aliphatic2	I, L, V		Mercapto	C
Amide	N, Q		Carboxamide	N, Q
		Monamino, Dicarboxylic		D, E
		Diamino, Monocarboxylic		H, K, R

##### Gapped amino acid composition

This feature represents the frequency of two amino acids (or amino acid groups) separated by x residues, x being the gap length. We experimented with gap lengths from 1 to 6.

#### Parameter selection and evaluation of SVM predictions

**Figure 2:**
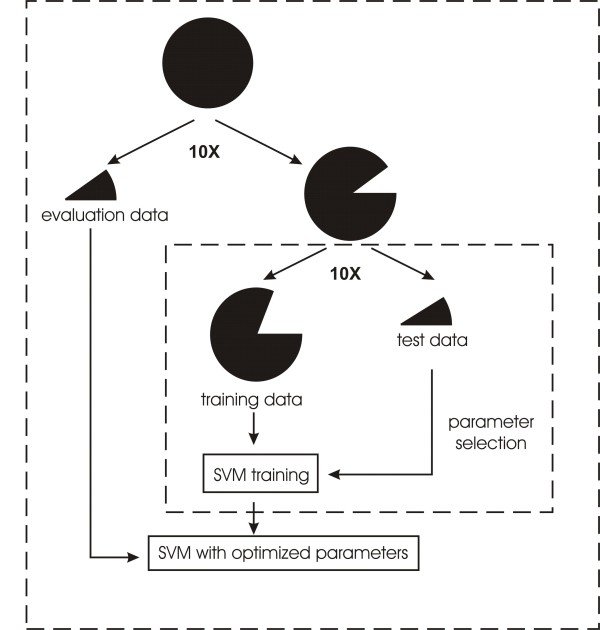
**Training and evaluation of SVM predictors**. The circle and pies indicate the dataset and portions thereof. The procedure in each dashed box was repeated ten times. The whole dataset was randomly divided into ten parts, with nine parts combined to construct the SVM model, and the remaining one to evaluate the model. The combined data for model construction were further divided randomly into ten subsets, in which nine subsets were combined to serve as training data, and the 10^th ^subset served as test data. See text for details.

In this study, we employed the SVM package LIBSVM [52], with the radial basis function (RBF) adopted as kernel function (K(x_i_, x_j_) = exp(-γ||x_i_-x_j_||^2^), γ>0), which requires the selection of the kernel parameter γ, and the penalty parameter C. To obtain the optimal parameters and to evaluate the predictions, we performed a 10-fold cross validation scheme for SVM parameter (C and γ) selection, followed by a 10-fold independent evaluation to assess the prediction performance (Figure 2). We first randomly divided the whole dataset into ten subsets of equal size. For each iteration of the ten rounds, nine subsets were combined to build SVM models, and the remaining subset was used for evaluation. The combined nine subsets were further subdivided into ten groups, whereof nine groups were combined and used to train SVM with given values for C and γ, while the remaining group was used to find the optimal combination of the two parameters. Finally, we assessed the performance of the selected C and γ values by using the evaluation data subset.

#### Performance evaluation

We calculated the overall accuracy for all classes combined, as well as the sensitivity (SN), specificity (SP), positive predictive value (PPV), and Matthews Correlation Coefficient (MCC) of each individual class as follows:

$$\text{Overall Accuracy }\left(\text{acc}\right)=\frac{\sum_{\text{i}=\text{1}}^{\text{n}}\text{TPi}}{\sum_{\text{i}=\text{1}}^{\text{n}}\text{(TPi}+\text{FNi})}*\text{1}00$$

i: the i-th class; n: total number of classes

For each class i:

$$\text{Sensitivity }\left(\text{SNi}\right)=\frac{\text{TPi}}{\text{TPi}+\text{FNi}}*100$$

$$\text{Specificity }\left(\text{SPi}\right)=\frac{\text{TNi}}{\text{TNi}+\text{FPi}}*100$$

$$\text{Positive predictive value }\left(\text{PPVi}\right)=\frac{\text{TPi}}{\text{TPi}+\text{FPi}}*100$$

$$\text{Matthews Correlation Coefficient }\left(\text{MCCi}\right)=\frac{\text{TPi*TNi}-\text{FPi*FNi}}{\sqrt{\text{(TPi}+\text{FPi)*(TPi}+\text{FNi)*(TNi}+\text{FPi)*(TNi}+\text{FNi)}}}$$

TP: true positives; FP: false positives; TN: true negatives; FN: false negatives

### Open reading frame (ORF) prediction for ESTs

Prot4EST [53] was used for the prediction of open reading frames (ORFs) in EST sequences. ESTs were first aligned with proteins from the NCBI non-redundant sequence database by BLASTX. The protein-EST alignment indicates the correct translation frame. For ESTs without significant BLAST matches, we used ESTScan [54] from within Prot4EST. ESTScan predicts ORFs based on a Hidden Markov Model (HMM) by recognizing the species-specific bias in hexanucleotide composition associated with coding and non-coding regions, and generates a matrix to represent this bias [54]. To generate the matrix, we trained ESTScan with all annotated *Arabidopsis *genomic and mRNA data collected from the European Molecular Biology Laboratory (EMBL) Nucleotide Sequence Database [55]. The mRNA data were mapped to genomic data in order to find the borders of coding/non-coding regions, which was needed to train the HMM. More details can be found in the publication describing EST-Scan [54].

## Results

We experimented with different sequence features to represent EST-peptides in SVM-based prediction of subcellular localization. As detailed in the Methods section, features included amino acid composition, grouped amino acid composition reflecting the physicochemical properties, gapped amino acid composition capturing the spatial context, as well as combinations thereof. The performance of the obtained prediction schemes varies considerably as shown below.

### Performance of predictors based on individual features

**Figure 3:**
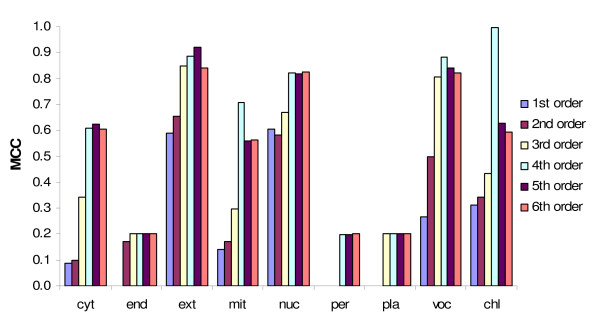
**Independent evaluation of SVM predictors based on different representations of amino acid composition**. The performance was assessed by the Matthews Correlation Coefficient (MCC). For most classes, the best MCC was obtained with the 4^th ^order amino acid composition (the frequency of tetra-peptides). Amino acid group-C and group-D composition yielded similar results (see Additional file 5).

Among the 41 sequence features investigated (Additional file 5), the best performance was obtained by the SVM predictor exploiting the 4^th ^order amino acid composition (Figure 3; Table 4, scheme 1), with a MCC >0.6 for all large classes (nuclear, cytosolic, mitochondrial, and chloroplastic locations; for sensitivity and positive predictive value, see Additional file 6). A similarly good performance was observed with SVM predictors based on the 6^th ^order group-C amino acid composition and the 7^th ^order group-D amino acid composition (Additional file 5). Unexpectedly, sequence features such as gapped amino acid composition and physicochemical properties represented by AAindices did not yield satisfying results, with MCCs for most classes below 0.4 (Additional file 5).

**Table 4:** Evaluation of results from top-ranking TESTLoc prediction schemes^1^

	Prediction scheme		chl	cyt	end	ext	mit	nuc	per	pla	vac
Expanded plant dataset	1. Top-performing individual feature (4^th ^order amino acid composition)	SN	99.9 (0.44)	**53 ** (12.2)	20 (42.2)	**83 ** (17.7)	**88.4 ** (7)	**82.7 ** (4.9)	20 (42.2)	20 (42.2)	**80 ** (23.3)
		PPV	99.9 (0.44)	76.1 (14.4)	20 (42.2)	96.3 (7.8)	67.9 (4.7)	88 (4.8)	20 (42.2)	20 (42.2)	**100 ** (0)
		MCC	0.99 (0.01)	0.61 (0.14)	0.2 (0.42)	**0.88 ** (0.1)	0.7 (0.07)	0.82 (0.04)	0.2 (0.42)	0.2 (0.42)	**0.88 ** (0.14)
	2. Integration of predictions from all sequence features	SN	100 (0)	45.5 (8.55)	20 (42.2)	69 (12)	86.1 (10.8)	78.1 (8.3)	40 (51.6)	**30 ** (48.3)	63.3 (24.6)
		PPV	99.3 (1.4)	**93.5 ** (8.6)	20 (42.2)	98 (6.3)	70.3 (11.6)	**97.4 ** (4.2)	11.7 (31.2)	8.8 (16.7)	100 (0)
		MCC	0.99 (0.01)	0.63 (0.08)	0.2 (0.42)	0.81 (0.07)	0.7 (0.05)	**0.85 ** (0.05)	0.16 (0.31)	0.15 (0.26)	0.78 (0.16)
	3. Integration attributions of all sequence features	SN	**100 ** (0)	9.3 (9.2)	10 (31.6)	48.5 (22.9)	82.2 (8.2)	80 (6.2)	0 (0)	0 (0)	0 (0)
		PPV	**100 ** (0)	28.8 (31.8)	10 (31.6)	77.8 (22)	53.1 (3.4)	76.2 (8.5)	0 (0)	0 (0)	0 (0)
		MCC	**1 ** (0)	0.1 (0.16)	0.1 (0.32)	0.6 (0.13)	0.5 (0.07)	0.7 (0.07)	0 (0)	0 (0)	0 (0)
	4. Integration of predictions from three top-performing features^2^	SN	99.9 (0.44)	50.5 (9.5)	**20 ** (42.2)	71 (12)	86.7 (17.7)	75.8 (7.9)	**50 ** (52.7)	30 (48.3)	63.3 (24.6)
		PPV	99.7 (0.6)	88.4 (9.2)	**20 ** (42.2)	**98 ** (6.3)	**71.2 ** (12.4)	96.1 (4.6)	**21.6 ** (41.4)	5 (8.1)	100 (0)
		MCC	0.99 (0.01)	0.65 (0.09)	**0.2 ** (0.42)	0.83 (0.07)	**0.71 ** (0.06)	0.82 (0.04)	**0.26 ** (0.4)	0.12 (0.19)	0.78 (0.16)
	5. Integration of attributes from three top-performing features	SN	94.4 (2.2)	52.2 (12.7)	20 (42.2)	75 (17.2)	84.2 (5.8)	77.7 (4.7)	20 (42.2)	20 (42.2)	76.7 (22.5)
		PPV	86.6 (3.7)	90.5 (11)	20 (42.2)	96 (8.4)	67.8 (3.7)	92 (4.3)	20 (42.2)	**20 ** (42.2)	100 (0)
		MCC	0.8 (0.05)	**0.66 ** (0.1)	0.2 (0.42)	0.84 (0.1)	0.68 (0.05)	0.81 (0.04)	0.2 (0.42)	**0.2 ** (0.42)	0.86 (0.14)

*Arabidopsis *validation dataset	Integration of predictions from three top-performing features	SN	47.4	58.5	80	100	89.8	90.2	0	100	100
		PPV	90.6	86.1	100	100	68.2	100	100	100	100
		MCC	0.42	0.67	0.89	1	0.57	0.94	0	1	1

^1 ^Numbers are the average of the 10-fold test. Numbers in parenthesis are the standard deviation. Bold numbers indicate the best values for each metric (SN, PPV, MCC) in each class of the expanded plant dataset. The values for SN and PPV are given in %. MCC, Matthews Correlation Coefficient. For other abbreviations, see footnote to Table 1.

^2 ^The three features are 4^th ^order amino acid composition, 6^th ^order group-C amino acid composition and 7^th ^order group-D amino acid composition

Three classes (plasma membrane, peroxisome and endoplasmic reticulum) are underrepresented, because data from these locations are still scarce. We included these classes because in practice, a query sequence could be from any of these locations. Were these locations left out, the predictor would inevitably misassign query sequences from these three classes. Yet, when these locations are included in the predictor, there is at least the possibility that the query sequence will be correctly assigned, even if the prediction accuracy may be low (20-50%). Eventually, this shortcoming will be alleviated when more data from these locations become available.

### Performance of predictors based on combined features

Previous studies showed that integration of multiple sequence features improves the performance of localization prediction [31,32,34]. We combined all the 41 sequence features described in the Methods section first by integrating attributes and second by integrating prediction results. To integrate attributes, the vectors of all sequence features were combined and used as input for SVM predictors. This type of integration yielded much lower performance than the best predictor based on a single feature (Table 4, scheme 3).

**Figure 4:**
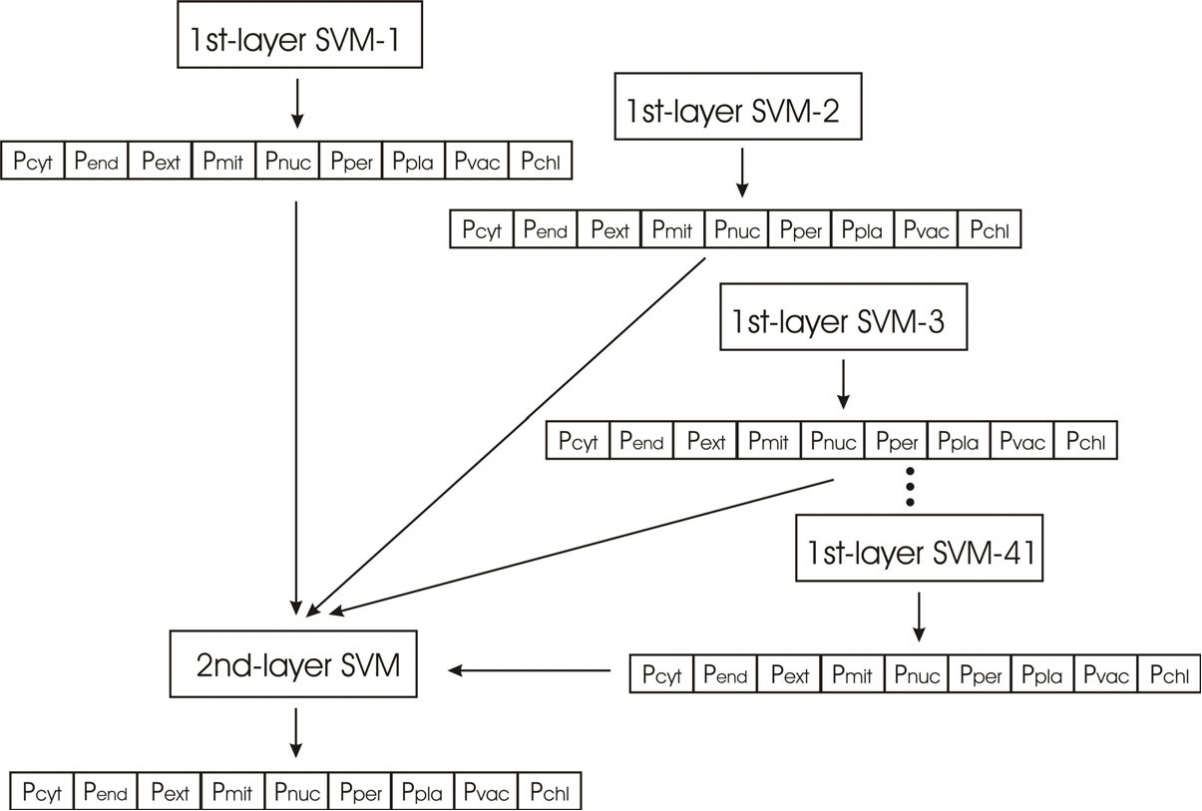
**Integration of predictions from SVM models based on individual features**. Each of the 41 SVM models built with single sequence features forms the first layer SVM and emits the probabilities for the query sequence to belong to the various classes. The probabilities are used as input for the second layer SVM.

Integration of prediction results from individual features was achieved by a two-layer SVM (Figure 4). The first layer consisted of SVM predictors based on a single sequence feature, yielding as output the probabilities of the query sequence to belong to each class. The outputs of all first-layer SVMs were combined and served as input for the second-layer SVM. Thus, each sequence was converted to a vector of size 369 (nine predictions for each of the 41 features). The two-layer SVM predictor showed similarly good performance as that based on the best single feature (Table 4, scheme 2).

In addition, we integrated the three features that yielded best performance (4^th ^order amino acid composition, 6^th ^order group-C composition and 7^th ^order group-D composition), as well as the predictions from these features. Again, these schemes showed a similar MCC as the predictors based on a single feature, but with lower sensitivity and higher positive predictive value for most classes (Table 4, scheme 4 and 5). Compared with the prediction from each of the three features individually, their integration often produced a much smaller rate of false positive predictions.

### ORF prediction from ESTs

Another challenge in EST-peptide-based localization prediction is the correct translation from nucleotide sequence. Unlike genomic data, ESTs often lack start codon and 5'-UTR, which otherwise help to detect the correct open reading frame (ORF). In addition, ESTs are products of single-pass reads often containing low quality regions with sequencing errors that further complicate the task. Several tools have been developed for ORF identification in EST sequences [3,54,56]. We chose Prot4EST [53], which combines similarity-based and machine-learning-based prediction of ORFs. The scoring matrix specific to *Arabidopsis *was obtained by training ESTScan with *Arabidopsis *data, as described in the Methods section. The accuracy of ORF prediction, evaluated by the percentage of correctly identified start/stop positions of coding regions, was over 70% (Additional file 7).

### Implementation of prediction methods and validation with *Arabidopsis *data

We built a tool named TESTLoc that combines EST translation with localization prediction. TESTLoc has two components. The first takes EST nucleotide sequences as input and generates EST-peptides via the tool Prot4EST. The second takes EST-peptides as input and outputs the probability that the peptide is targeted to a given subcellular compartment. The current model predicts nine locations: cytosol, endoplasmic reticulum, extracellular space, mitochondria, nucleus, peroxisomes, plasma membrane, vacuole, and chloroplasts.

To evaluate the combined prediction of ORFs and subcellular localization, we tested the performance of TESTLoc with *Arabidopsis *ESTs that correspond to proteins of known localization. Sequences were represented by three best-performing features: 4^th ^order amino acid composition, 6^th ^order group-C amino acid composition, and 7^th ^order group-D amino acid composition. The resulting predictions showed high MCC values (>0.6) for most classes (Table 4).

TESTLoc can be downloaded and executed locally. The sequence features to use and how to combine them can be chosen by users via editing the configuration file. It should be emphasized that TESTLoc allows users to train new models with their own data, which facilitates the analysis of sequences from other taxonomic groups or individual species. (Should EST training data be scarce, artificial EST-peptides can be generated by breaking up full-length protein sequences as devised here.) Note that TESTLoc is designed for EST data, and should not be applied to full-length proteins (Additional file 8).

## Discussion

### Effects of various sequence features

When experimenting with different kinds of n^th ^order amino acid composition, we observed a common trend: for the training data, the performance improved with increasing order until reaching a peak; for the evaluation data, at first the performance improved with the order, reached a peak, and then dropped again. This shows that higher-order composition made the scheme remember the instances in the training procedure, a phenomenon called overfitting. Therefore, we did not experiment with orders higher than six for ungrouped amino acid composition, and eight for grouped amino acid composition.

### Localization signals in partial sequences

The sequence signals that guide the sorting of proteins into different subcellular compartments are not well understood. For a given compartment, more than one targeting signal seem to exist. Apparently, peroxisomal targeting involves at least three different signals, and mitochondrial targeting involves four [57,58]. Although characterized targeting signals are generally short N- or C-terminal peptide motifs, in many cases signals appear to be embedded in the central region of the protein. This explains why EST-peptides, which often lack the N-terminal portion of a protein, are still information-rich enough for inferring subcellular localization, as we have demonstrated here.

In our study, tetra-peptides (4^th ^order amino acid composition) yielded the best performance. This is unexpected for a feature space two orders of magnitude larger (20^4 ^= 160,000) than the sample size (~1,500 sequences containing a total of ~300,000 tetra-peptides). Statistically, most tetra-peptides should be represented by only a few proteins, which should render machine learning rather ineffective. Alternatively, certain tetra-peptides may be over-represented in a given class, either due to a strong location-related signal or an artifact arising from redundancy in the dataset. To clarify the situation, we scrutinized the tetra-peptides present in our data (Additional file 9).

The size of the dataset allows for a total of 318,823 tetra-peptides. While the upper limit of distinct tetra-peptides (motifs) is 160,000, our particular dataset contains two third (99,107). About one half (45,883) of the occurring motifs are found in single classes and are present in only a few members of a given class (<3%). Further, the motifs within a class do not show conserved sequence patterns. The complementary trend applies to the absence of tetra-peptides: no particular tetra-peptide is absent from only one class. In sum, there is no notable enrichment of, or bias against, certain tetra-peptide motifs in a class, nor any sign of a data redundancy artifact.

How then does this feature category achieve superior prediction performance? One possibility is that tetra-peptides bear targeting information in the form of more complex patterns such as nonadjacent correlation of multiple tetra-peptides, which we would not have recognized in our analysis.

## Conclusions

Our results described here show that the SVM machine learning method, together with sequence features carefully chosen, predicts the subcellular location of EST-derived proteins with high accuracy, thus filling the need for a tool tailored to EST data. We implemented TESTLoc as a fully automated pipeline combining EST-ORF prediction and localization prediction. This tool opens new avenues for systematic analysis of EST data from any eukaryote including challenging photosynthetic taxa such as plants.

## Authors' contributions

YQS developed and implemented the methods. GB conceived of the study, participated in its design, and supervised the process. YQS drafted the manuscript. Both authors read and approved the final manuscript.

## Supplementary Material

Additional file 1 **Influence of sequence similarity on the accuracy of localization prediction by TESTLoc and BLAST**. Click here for file

Additional file 2 **List of available subcellular localization prediction methods**. Click here for file

Additional file 3 **Gene Ontology (GO) term of proteins from the plant dataset used in this study**. Click here for file

Additional file 4 **Selection of *Arabidopsis *ESTs corresponding to proteins of known localization**. Click here for file

Additional file 5 **Performance of predicting subcellular localization of EST-derived proteins, based on each sequence feature (including amino acid composition, grouped amino acid composition, gapped amino acid composition, and AAindex)**. Click here for file

Additional file 6 **The independent evaluation of SVM predictors based on different representations of amino acid composition, measured with sensitivity and positive predictive value**. Click here for file

Additional file 7 **Accuracy of ESTScan for the prediction of start/stop positions of coding regions in EST sequences**. Click here for file

Additional file 8 **Comparison of prediction performance of available tools and TESTLoc on full-length plant protein sequences**. Click here for file

Additional file 9 **Tetra-peptides present in the expanded plant ESTs data**. Click here for file
