# Supplementary material for: TESTLoc: protein subcellular localization prediction from EST data
Source: BMC Bioinformatics. 2010 Nov 15;11:563. doi: 10.1186/1471-2105-11-563 (PMC3000424; doi:10.1186/1471-2105-11-563)
Supplement: Additional file 1 — Influence of sequence similarity on the accuracy of localization prediction by TESTLoc and BLAST. [file 1471-2105-11-563-S1.PDF]

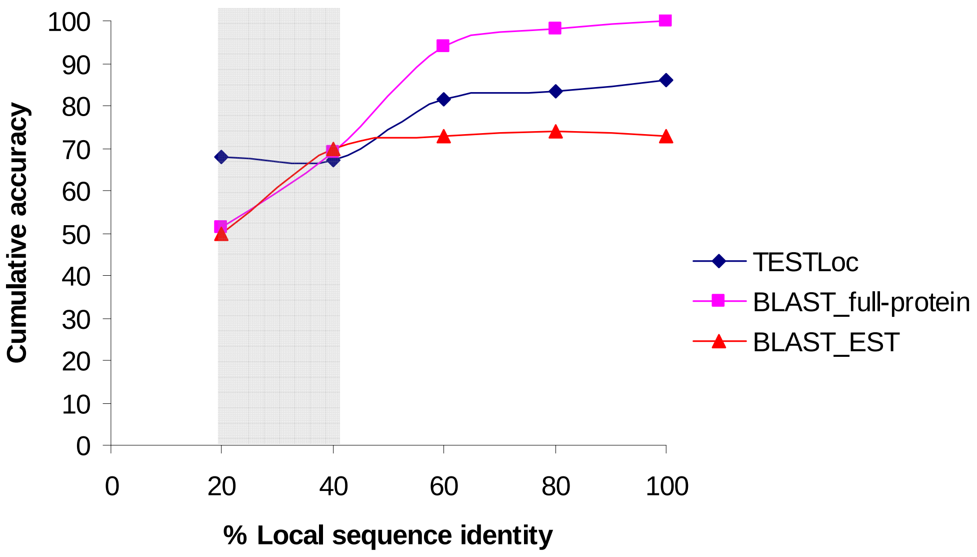

Additional file 1. Influence of sequence similarity on the accuracy of protein localization predicted by TESTLoc and inferred via BLAST. Sequence similarity was measured as percent local sequence identity calculated from BLAST alignment (number of identical residues/number of aligned residues). Purple squares, accuracy of BLAST-based localization prediction of full-length proteins. The dataset, taken from [8], contains proteins from SWISSPROT. The subcellular location annotation of the top hit sequence is transferred to the query sequence and then compared to the known location of the query sequence. Orange triangles: accuracy of BLAST-based localization prediction of EST-peptides. Using the data collected in this report, EST-peptides are blasted against full-length proteins (excluding the proteins corresponding to the query ESTs), and the location annotation is transferred from the aligned full-length protein. Blue diamonds, accuracy of TESTLoc versus the sequence similarity between the test and training EST-peptides collected in this study. It is obvious that BLAST is less effective in localization prediction of EST-derived peptides than TESTLoc, especially for ESTs with low similarity to known proteins (the shaded area).
