# Supplementary material for: TESTLoc: protein subcellular localization prediction from EST data
Source: BMC Bioinformatics. 2010 Nov 15;11:563. doi: 10.1186/1471-2105-11-563 (PMC3000424; doi:10.1186/1471-2105-11-563)
Supplement: Additional file 2 — List of available subcellular localization prediction methods. [file 1471-2105-11-563-S2.DOC]

Additional file 2. List of subcellular localization prediction methods

| Name of the predictor or author | Sequence feature | Computational methods |
| --- | --- | --- |
| PA-SUB | Sequence similarity and text annotation | Naïve Bayes |
| EpiLoc |  | Support Vector Machine |
| ProLoc-GO | Gene Ontology term | Genetic algorithm based method combined with SVM |
| PSLT | InterPro domains and specific membrane domains | Likelihood calculated by Bayes' rule |
| Predotar | Targeting peptide | Neural network |
| TargetP |  | Neural network |
| iPSORT |  | Alphabet indexing and pattern rule |
| Protein prowler |  | Neural network |
| pSLIP | Physicochemical properties | Support Vector Machine |
| Subloc | Amino acid composition | Support Vector Machine |
| NNPSL |  | Neural network |
| LOCSVMPSI | Position-specific scoring matrix and amino acid composition of four segments | Support Vector Machine |
| ESLpred | Amino acid composition, 33 physicochemical properties, dipeptide composition, PSI-blast result, and  combined feature of the above | Support Vector Machine |
| Cai et al | Amino acid composition, quasi-sequence-order (up to 13 gaps), and physicochemical properties (hydrophobicity, hydrophilicity, side-chain volume) | Support Vector Machine |
| LOCtree | Evolutionary profiles, global amino acid composition,  50N-terminal amino acid composition, amino acid composition in three secondary structure states,  and the output of signalP | Support Vector Machine |
| Yuan | Amino acid composition and paired amino acid composition | Hidden Markov Model |
| PSORTII | A set of sequence-derived features | K Nearest Neighbors |
| WoLF PSORT | Features from iPSORT and PSORTII, together with amino acid content | Weighted K Nearest Neighbors |
| Gao, et al | Amino acid composition, dipeptide composition, and physicochemical properties | K Nearest Neighbors |
| MITOPRED | Pfam domains occurrence, amino acid composition, and PI value | Score of different features |
| pTARGET | Pfam domains occurrence and amino acid composition | Score of different features |
| MitoProt | Targeting sequence and hydrophobicity characteristics | Multivariate analysis |
| SherLoc | Amino acid composition, targeting signals, motif, and text search | Support Vector Machine |
| MultiLoc | Amino acid composition, targeting signals, phylogenetic profiles, motif, and GO term | Support Vector Machine |
| PSLDoc | Gapped dipeptide and position specific scoring matrix | Support Vector Machine |
| KnowPred | Sequence similarity | Similarity score |
| YLoc | Amino acid composition, normalized amino acid composition, pseudo-amino acid composition, grouped amino acid composition, PROSITE patterns and GO terms | Naïve Bayes |

* As new tools emerge each year, this list is not exhaustive and includes the representative tools
