# Supplementary material for: TESTLoc: protein subcellular localization prediction from EST data
Source: BMC Bioinformatics. 2010 Nov 15;11:563. doi: 10.1186/1471-2105-11-563 (PMC3000424; doi:10.1186/1471-2105-11-563)
Supplement: Additional file 3 — Gene Ontology (GO) term of proteins from the plant dataset used in this study. [file 1471-2105-11-563-S3.DOC]

The remaining categories are not listed

The remaining categories are not listed

Additional file 3. Gene Ontology (GO) term of proteins from the plant dataset used in this study. The GO term was obtained from the SwissProt annotation of the corresponding sequences. The categories are ordered according to their abundance. Altogether, 173 categories of biological process and 183 categories of molecular function are found. Not all categories are listed in the legend due to space limitation.
