# Supplementary material for: TESTLoc: protein subcellular localization prediction from EST data
Source: BMC Bioinformatics. 2010 Nov 15;11:563. doi: 10.1186/1471-2105-11-563 (PMC3000424; doi:10.1186/1471-2105-11-563)
Supplement: Additional file 4 — Selection of Arabidopsis ESTs corresponding to proteins of known localization. [file 1471-2105-11-563-S4.DOC]

*Arabidopsis* proteins

from SWISSPROT (1,035 sequences)

*Arabidopsis* ESTs from dbEST

(420,789 sequences)

BLASTX

Translate EST

Remove redundancy

*Arabidopsis* dataset

(386 ESTs)

>90% of total length

Identity >90%

**Protein**

**EST-ORF**

…AGTHTKLPQEC…

…AGTHSKLPQAC…

**Additional file 4**. Selection of *Arabidopsis* ESTs corresponding to proteins of known localization. If the aligned region of an EST is longer than 90% of its total length, and the amino acid identity between the protein and the translated EST is over 90%, the EST-protein pair is regarded as derived from the same gene. The thus selected ESTs were translated into amino acid sequences in the frame indicated by BLASTX alignment. Sequence redundancy was reduced by CD-hit so that no pair of sequences shares more than 60% similarity. The final dataset is composed of ESTs instead of translated amino acid sequences.
