# Supplementary material for: TESTLoc: protein subcellular localization prediction from EST data
Source: BMC Bioinformatics. 2010 Nov 15;11:563. doi: 10.1186/1471-2105-11-563 (PMC3000424; doi:10.1186/1471-2105-11-563)
Supplement: Additional file 5 — Performance of predicting subcellular localization of EST-derived proteins, based on each sequence feature (including amino acid composition, grouped amino acid composition, gapped amino acid composition, and AAindex). [file 1471-2105-11-563-S5.DOC]

**Additional file 5. Performance of predicting subcellular localization of EST-derived proteins,** based on each sequence feature (Numbers are averaged by 10-round evaluation)

| **Matthews correlation coefficient value** | | | | | | | | | |
| --- | --- | --- | --- | --- | --- | --- | --- | --- | --- |
| natural amino acid composition | | | | | | | | | |
|  | cyt^1^ | end | ext | mit | nuc | per | pla | voc | chl |
| 1st order | 0.09 | 0.00 | 0.59 | 0.14 | 0.60 | 0.00 | 0.00 | 0.27 | 0.31 |
| 2nd order | 0.10 | 0.17 | 0.65 | 0.17 | 0.58 | 0.00 | 0.00 | 0.50 | 0.34 |
| 3rd order | 0.34 | 0.20 | 0.85 | 0.30 | 0.67 | 0.00 | **0.20** | 0.80 | 0.43 |
| 4th order | 0.61 | 0.20 | 0.88 | 0.71 | **0.82** | **0.20** | **0.20** | **0.88** | **1.00** |
| 5th order | 0.62 | 0.20 | **0.92** | 0.56 | **0.82** | **0.20** | **0.20** | 0.84 | 0.63 |
| 6th order | 0.60 | 0.20 | 0.84 | 0.56 | **0.82** | **0.20** | **0.20** | 0.82 | 0.59 |
| group C amino acid composition | | | | | | | | | |
|  | cyt | end | ext | mit | nuc | per | pla | voc | chl |
| 1st order | 0.00 | 0.20 | 0.34 | 0.07 | 0.54 | 0.00 | 0.00 | 0.00 | 0.25 |
| 2nd order | 0.04 | 0.00 | 0.41 | 0.09 | 0.54 | 0.00 | 0.00 | 0.46 | 0.28 |
| 3rd order | 0.03 | 0.10 | 0.57 | 0.19 | 0.54 | 0.00 | 0.00 | 0.66 | 0.32 |
| 4th order | 0.31 | 0.20 | 0.66 | 0.55 | 0.63 | 0.00 | 0.00 | 0.78 | **1.00** |
| 5th order | 0.52 | 0.20 | 0.81 | 0.64 | 0.72 | **0.20** | **0.20** | 0.78 | **1.00** |
| 6th order | 0.65 | 0.20 | 0.77 | **0.72** | 0.77 | **0.20** | **0.20** | 0.84 | 0.99 |
| 7th order | **0.67**^2^ | 0.20 | 0.87 | 0.69 | 0.79 | **0.20** | **0.20** | 0.84 | **1.00** |
| 8th order | 0.61 | 0.20 | 0.87 | 0.53 | 0.79 | 0.10 | **0.20** | 0.84 | 0.58 |
| group D amino acid composition | | | | | | | | | |
|  | cyt | end | ext | mit | nuc | per | pla | voc | chl |
| 1st order | -0.01 | 0.17 | 0.49 | 0.10 | 0.52 | 0.00 | 0.00 | 0.06 | 0.25 |
| 2nd order | 0.04 | 0.00 | 0.51 | 0.11 | 0.55 | 0.00 | 0.00 | 0.62 | 0.29 |
| 3rd order | 0.07 | 0.10 | 0.71 | 0.15 | 0.53 | 0.00 | 0.00 | 0.71 | 0.31 |
| 4th order | 0.33 | 0.20 | 0.85 | 0.58 | 0.67 | 0.00 | **0.20** | 0.80 | **1.00** |
| 5th order | 0.54 | 0.20 | 0.86 | 0.60 | 0.71 | **0.20** | **0.20** | 0.86 | 0.92 |
| 6th order | 0.61 | 0.20 | 0.85 | 0.71 | 0.78 | **0.20** | **0.20** | 0.86 | **1.00** |
| 7th order | 0.65 | 0.20 | 0.81 | **0.72** | 0.80 | **0.20** | **0.20** | 0.84 | **1.00** |
| 8th order | 0.59 | 0.20 | 0.80 | 0.54 | 0.78 | 0.10 | **0.20** | 0.84 | 0.58 |
| natural amino acid | | | | | | | | | |
|  | cyt | end | ext | mit | nuc | per | pla | voc | chl |
| one gap | 0.19 | 0.20 | 0.63 | 0.53 | 0.67 | 0.00 | 0.00 | 0.48 | **1.00** |
| two gap | 0.16 | 0.34 | 0.76 | 0.53 | 0.63 | 0.00 | 0.00 | 0.42 | **1.00** |
| three gap | 0.15 | **0.37** | 0.72 | 0.53 | 0.65 | 0.00 | 0.00 | 0.53 | **1.00** |
| four gap | 0.13 | 0.20 | 0.52 | 0.51 | 0.64 | 0.00 | 0.00 | 0.39 | **1.00** |
| five gap | 0.14 | 0.20 | 0.55 | 0.49 | 0.63 | 0.00 | 0.00 | 0.49 | 0.98 |
| six gap | 0.09 | **0.37** | 0.69 | 0.54 | 0.61 | 0.00 | 0.00 | 0.55 | 0.98 |
| group C amino acid composition | | | | | | | | | |
|  | cyt | end | ext | mit | nuc | per | pla | voc | chl |
| one gap | 0.03 | 0.00 | 0.34 | 0.07 | 0.49 | 0.00 | 0.00 | 0.05 | 0.23 |
| two gap | 0.00 | 0.00 | 0.53 | 0.04 | 0.50 | 0.00 | 0.00 | 0.00 | 0.24 |
| three gap | -0.02 | 0.10 | 0.39 | 0.05 | 0.51 | 0.00 | 0.00 | 0.00 | 0.26 |
| four gap | 0.00 | 0.00 | 0.40 | 0.07 | 0.52 | 0.00 | 0.00 | 0.00 | 0.26 |
| five gap | 0.00 | 0.00 | 0.44 | 0.03 | 0.50 | 0.00 | 0.00 | 0.00 | 0.25 |
| six gap | 0.00 | 0.00 | 0.50 | 0.10 | 0.47 | 0.00 | 0.00 | 0.00 | 0.27 |
| group D amino acid composition | | | | | | | | | |
|  | cyt | end | ext | mit | nuc | per | pla | voc | chl |
| one gap | 0.03 | 0.00 | 0.54 | 0.10 | 0.51 | 0.00 | 0.00 | 0.29 | 0.27 |
| two gap | 0.01 | 0.00 | 0.65 | 0.08 | 0.51 | 0.00 | 0.00 | 0.00 | 0.26 |
| three gap | 0.04 | 0.20 | 0.58 | 0.03 | 0.47 | 0.00 | 0.00 | 0.00 | 0.24 |
| four gap | 0.00 | 0.10 | 0.49 | 0.06 | 0.50 | 0.00 | 0.00 | 0.00 | 0.26 |
| five gap | 0.00 | 0.00 | 0.53 | 0.03 | 0.47 | 0.00 | 0.00 | 0.17 | 0.25 |
| six gap | 0.01 | 0.00 | 0.54 | 0.18 | 0.44 | 0.00 | 0.00 | 0.17 | 0.27 |
|  | | | | | | | | | |
| aaindex | 0.18 | 0.30 | 0.64 | 0.58 | 0.70 | 0.00 | 0.00 | 0.30 | 0.99 |
|  | | | | | | | | | |
| **Sensitivity (%)** | | | | | | | | | |
| natural amino acid composition | | | | | | | | | |
|  | cyt | end | ext | mit | nuc | per | pla | voc | chl |
| 1st order | 5.8 | 0.0 | 48.5 | 20.1 | 65.8 | 0.0 | 0.0 | 16.7 | 89.2 |
| 2nd order | 5.8 | 20.0 | 56.5 | 22.7 | 60.8 | 0.0 | 0.0 | 40.0 | 89.4 |
| 3rd order | 28.6 | 20.0 | 79.0 | 36.9 | 72.3 | 0.0 | **20.0** | 70.0 | 83.8 |
| 4th order | 53.0 | 20.0 | 83.0 | 88.4 | **82.7** | **20.0** | **20.0** | **80.0** | 99.9 |
| 5th order | 49.6 | 20.0 | **87.5** | 58.6 | 78.1 | **20.0** | **20.0** | 73.3 | 92.5 |
| 6th order | 46.3 | 20.0 | 73.5 | 49.9 | 75.0 | **20.0** | **20.0** | 70.0 | 96.0 |
| group C amino acid composition | | | | | | | | | |
|  | cyt | end | ext | mit | nuc | per | pla | voc | chl |
| 1st order | 0.0 | 20.0 | 22.0 | 13.6 | 52.7 | 0.0 | 0.0 | 0.0 | 92.2 |
| 2nd order | 1.7 | 0.0 | 30.0 | 16.2 | 55.8 | 0.0 | 0.0 | 33.3 | 90.9 |
| 3rd order | 1.7 | 10.0 | 48.5 | 25.2 | 57.7 | 0.0 | 0.0 | 46.7 | 88.9 |
| 4th order | 20.2 | 20.0 | 58.5 | 78.6 | 71.2 | 0.0 | 0.0 | 66.7 | **100.0** |
| 5th order | 43.8 | 20.0 | 77.0 | 82.5 | 77.3 | **20.0** | **20.0** | 63.3 | **100.0** |
| 6th order | 53.0 | 20.0 | 70.5 | 90.6 | 79.2 | **20.0** | **20.0** | 73.3 | 99.6 |
| 7th order | **54.7** | 20.0 | 79.0 | 90.9 | 76.9 | **20.0** | **20.0** | 73.3 | 99.9 |
| 8th order | 48.8 | 20.0 | 79.5 | 51.8 | 71.5 | 10.0 | **20.0** | 73.3 | 93.5 |
| group D amino acid composition | | | | | | | | | |
|  | cyt | end | ext | mit | nuc | per | pla | voc | chl |
| 1st order | 0.0 | 20.0 | 34.0 | 17.2 | 50.4 | 0.0 | 0.0 | 3.3 | 91.0 |
| 2nd order | 2.5 | 0.0 | 40.0 | 13.6 | 56.9 | 0.0 | 0.0 | 53.3 | 91.3 |
| 3rd order | 4.2 | 10.0 | 65.0 | 22.3 | 56.2 | 0.0 | 0.0 | 53.3 | 88.1 |
| 4th order | 29.6 | 20.0 | 81.0 | 76.4 | 74.2 | 0.0 | **20.0** | 66.7 | **100.0** |
| 5th order | 44.8 | 20.0 | 83.0 | 79.3 | 77.7 | **20.0** | **20.0** | 76.7 | 95.1 |
| 6th order | 48.9 | 20.0 | 76.5 | 89.3 | 80.8 | **20.0** | **20.0** | 76.7 | **100.0** |
| 7th order | 53.9 | 20.0 | 69.0 | **95.5** | 74.6 | **20.0** | **20.0** | 73.3 | 99.9 |
| 8th order | 44.6 | 20.0 | 69.0 | 49.5 | 71.9 | 10.0 | **20.0** | 73.3 | 95.0 |
| natural amino acid composition | | | | | | | | | |
|  | cyt | end | ext | mit | nuc | per | pla | voc | chl |
| one gap | 15.2 | 20.0 | 60.5 | 75.1 | 76.2 | 0.0 | 0.0 | 33.3 | **100.0** |
| two gap | 8.4 | **40.0** | 66.5 | 79.0 | 72.3 | 0.0 | 0.0 | 26.7 | 99.9 |
| three gap | 16.0 | **40.0** | 71.0 | 72.8 | 75.4 | 0.0 | 0.0 | 40.0 | 99.7 |
| four gap | 10.8 | 20.0 | 46.5 | 76.4 | 72.7 | 0.0 | 0.0 | 26.7 | 99.6 |
| five gap | 10.8 | 20.0 | 44.5 | 76.0 | 69.6 | 0.0 | 0.0 | 33.3 | 98.2 |
| six gap | 7.5 | **40.0** | 62.5 | 81.2 | 66.5 | 0.0 | 0.0 | 46.7 | 98.5 |
| group C amino acid composition | | | | | | | | | |
|  | cyt | end | ext | mit | nuc | per | pla | voc | chl |
| one gap | 1.7 | 0.0 | 28.0 | 12.6 | 52.3 | 0.0 | 0.0 | 3.3 | 90.6 |
| two gap | 0.0 | 0.0 | 42.0 | 13.0 | 53.8 | 0.0 | 0.0 | 0.0 | 90.2 |
| three gap | 0.0 | 10.0 | 32.5 | 10.7 | 53.5 | 0.0 | 0.0 | 0.0 | 92.2 |
| four gap | 0.0 | 0.0 | 30.0 | 12.6 | 56.2 | 0.0 | 0.0 | 0.0 | 91.3 |
| five gap | 0.0 | 0.0 | 28.0 | 5.8 | 54.6 | 0.0 | 0.0 | 0.0 | 93.4 |
| six gap | 0.0 | 0.0 | 34.0 | 13.3 | 53.1 | 0.0 | 0.0 | 0.0 | 92.0 |
| group D amino acid composition | | | | | | | | | |
|  | cyt | end | ext | mit | nuc | per | pla | voc | chl |
| one gap | 2.5 | 0.0 | 46.0 | 15.6 | 53.5 | 0.0 | 0.0 | 16.7 | 90.1 |
| two gap | 0.8 | 0.0 | 56.5 | 14.3 | 56.2 | 0.0 | 0.0 | 0.0 | 89.9 |
| three gap | 2.5 | 20.0 | 48.5 | 10.0 | 51.9 | 0.0 | 0.0 | 0.0 | 90.7 |
| four gap | 0.0 | 10.0 | 38.5 | 12.6 | 54.6 | 0.0 | 0.0 | 0.0 | 91.2 |
| five gap | 0.0 | 0.0 | 38.0 | 5.8 | 51.5 | 0.0 | 0.0 | 10.0 | 93.5 |
| six gap | 0.8 | 0.0 | 40.0 | 19.7 | 48.9 | 0.0 | 0.0 | 10.0 | 91.3 |
|  | | | | | | | | | |
| aaindex | 15.1 | 30.0 | 60.5 | 79.3 | 78.5 | 0.0 | 0.0 | 23.3 | **100.0** |
|  | | | | | | | | | |
| **Specificity (%)** | | | | | | | | | |
| natural amino acid composition | | | | | | | | | |
|  | cyt | end | ext | mit | nuc | per | pla | voc | chl |
| 1st order | 98.7 | 100.0 | 99.1 | 90.9 | 92.1 | 100.0 | 100.0 | 99.9 | 38.0 |
| 2nd order | 99.0 | 99.9 | 99.2 | 90.6 | 93.3 | 100.0 | 99.9 | 99.6 | 40.7 |
| 3rd order | 96.9 | 100.0 | 99.7 | 89.1 | 93.2 | 100.0 | 100.0 | 99.9 | 58.0 |
| 4th order | 98.5 | 100.0 | 99.8 | 89.1 | 97.4 | 99.9 | 100.0 | 100.0 | 99.8 |
| 5th order | 99.0 | 100.0 | 99.9 | 93.0 | 98.4 | 99.9 | 100.0 | 100.0 | 69.2 |
| 6th order | 99.2 | 100.0 | 99.9 | 96.5 | 99.5 | 100.0 | 100.0 | 100.0 | 60.7 |
| group C amino acid composition | | | | | | | | | |
|  | cyt | end | ext | mit | nuc | per | pla | voc | chl |
| 1st order | **100.0** | 99.9 | 99.6 | 91.7 | 94.7 | 100.0 | 100.0 | 100.0 | 26.9 |
| 2nd order | 99.8 | 100.0 | 99.1 | 90.7 | 93.2 | 100.0 | 100.0 | 99.3 | 32.0 |
| 3rd order | 98.9 | 100.0 | 98.9 | 90.0 | 92.5 | 100.0 | 100.0 | 100.0 | 39.3 |
| 4th order | 98.6 | 100.0 | 99.3 | 83.4 | 92.7 | 100.0 | 100.0 | 99.9 | 100.0 |
| 5th order | 98.3 | 100.0 | 99.5 | 87.3 | 94.9 | 100.0 | 100.0 | 100.0 | 100.0 |
| 6th order | 99.2 | 100.0 | 99.6 | 88.8 | 96.4 | 99.9 | 100.0 | 100.0 | 99.1 |
| 7th order | 99.2 | 100.0 | 99.9 | 87.0 | 97.6 | 99.9 | 100.0 | 100.0 | 99.8 |
| 8th order | 98.8 | 100.0 | 99.9 | 94.1 | 99.2 | 99.9 | 100.0 | 100.0 | 62.2 |
| group D amino acid composition | | | | | | | | | |
|  | cyt | end | ext | mit | nuc | per | pla | voc | chl |
| 1st order | 99.8 | 99.6 | 99.3 | 90.4 | 94.6 | 100.0 | 100.0 | 99.9 | 28.3 |
| 2nd order | 99.2 | 100.0 | 99.3 | 93.6 | 93.3 | 100.0 | 100.0 | 99.7 | 32.1 |
| 3rd order | 98.7 | 100.0 | 99.1 | 89.8 | 92.6 | 100.0 | 100.0 | 100.0 | 39.3 |
| 4th order | 97.0 | 100.0 | 99.7 | 86.3 | 93.3 | 100.0 | 100.0 | 100.0 | 100.0 |
| 5th order | 98.5 | 100.0 | 99.6 | 86.4 | 94.3 | 99.9 | 100.0 | 100.0 | 96.6 |
| 6th order | 99.1 | 100.0 | 99.9 | 88.4 | 96.5 | 100.0 | 100.0 | 100.0 | 99.5 |
| 7th order | 99.1 | 100.0 | 99.9 | 86.4 | 98.6 | 99.9 | 100.0 | 100.0 | 99.8 |
| 8th order | 99.2 | 100.0 | 99.9 | 95.5 | 98.7 | 99.9 | 100.0 | 100.0 | 59.9 |
| natural amino acid composition | | | | | | | | | |
|  | cyt | end | ext | mit | nuc | per | pla | voc | chl |
| one gap | 97.3 | 99.8 | 98.9 | 83.2 | 92.4 | 100.0 | 100.0 | 99.8 | 100.0 |
| two gap | 98.8 | 99.7 | 99.7 | 81.0 | 92.2 | 100.0 | 99.9 | 99.8 | 100.0 |
| three gap | 96.3 | 99.9 | 99.0 | 84.8 | 91.9 | 100.0 | 99.9 | 99.9 | 100.0 |
| four gap | 97.6 | 100.0 | 99.0 | 80.9 | 92.3 | 100.0 | 99.9 | 99.8 | 100.0 |
| five gap | 97.8 | 100.0 | 99.2 | 79.9 | 92.8 | 100.0 | 99.9 | 99.5 | 100.0 |
| six gap | 98.6 | 99.7 | 99.3 | 80.4 | 93.0 | 100.0 | 99.9 | 99.5 | 100.0 |
| group C amino acid composition | | | | | | | | | |
|  | cyt | end | ext | mit | nuc | per | pla | voc | chl |
| one gap | 99.9 | 100.0 | 98.6 | 92.3 | 91.7 | 100.0 | 100.0 | 99.8 | 27.4 |
| two gap | 99.9 | 100.0 | 99.3 | 90.0 | 91.6 | 100.0 | 100.0 | 99.9 | 29.3 |
| three gap | 99.1 | 99.8 | 98.7 | 92.8 | 92.6 | 100.0 | 100.0 | 99.8 | 28.1 |
| four gap | 99.9 | 100.0 | 99.3 | 92.1 | 92.0 | 100.0 | 100.0 | 100.0 | 28.9 |
| five gap | 100.0 | 100.0 | 99.5 | 95.8 | 91.3 | 100.0 | 100.0 | 100.0 | 25.1 |
| six gap | 100.0 | 100.0 | 99.5 | 92.8 | 90.2 | 100.0 | 100.0 | 100.0 | 28.9 |
| group D amino acid composition | | | | | | | | | |
|  | cyt | end | ext | mit | nuc | per | pla | voc | chl |
| one gap | 99.0 | 100.0 | 99.2 | 91.3 | 92.4 | 100.0 | 100.0 | 100.0 | 31.6 |
| two gap | 99.7 | 100.0 | 99.1 | 91.5 | 91.4 | 100.0 | 100.0 | 100.0 | 31.1 |
| three gap | 99.3 | 100.0 | 99.2 | 91.3 | 91.1 | 100.0 | 100.0 | 99.8 | 28.6 |
| four gap | 100.0 | 100.0 | 99.5 | 91.4 | 91.3 | 100.0 | 100.0 | 100.0 | 28.8 |
| five gap | 100.0 | 100.0 | 99.5 | 95.6 | 91.0 | 100.0 | 99.8 | 100.0 | 25.2 |
| six gap | 99.8 | 100.0 | 99.6 | 92.5 | 90.7 | 100.0 | 100.0 | 100.0 | 30.4 |
|  | | | | | | | | | |
| aaindex | 97.2 | 100.0 | 98.9 | 84.6 | 93.3 | 100.0 | 100.0 | 99.4 | 99.3 |
|  | | | | | | | | | |
| positive predictive value (%) | | | | | | | | | |
| natural amino acid composition | | | | | | | | | |
|  | cyt | end | ext | mit | nuc | per | pla | voc | chl |
| 1st order | 25.0 | 0.0 | 80.6 | 41.2 | 73.8 | 0.0 | 0.0 | 45.0 | 57.2 |
| 2nd order | 29.2 | 15.0 | 80.0 | 44.7 | 74.9 | 0.0 | 0.0 | 67.5 | 58.5 |
| 3rd order | 55.7 | 20.0 | 92.7 | 52.0 | 76.6 | 0.0 | 20.0 | 96.7 | 65.2 |
| 4th order | 76.1 | 20.0 | 96.3 | 67.9 | 88.0 | 20.0 | 20.0 | 100.0 | 99.9 |
| 5th order | 85.8 | 20.0 | 98.0 | 72.3 | 92.8 | 20.0 | 20.0 | 100.0 | 72.7 |
| 6th order | 86.6 | 20.0 | 97.5 | 81.9 | 97.7 | 20.0 | 20.0 | 100.0 | 67.5 |
| group C amino acid composition | | | | | | | | | |
|  | cyt | end | ext | mit | nuc | per | pla | voc | chl |
| 1st order | 0.0 | 20.0 | 60.0 | 35.1 | 77.5 | 0.0 | 0.0 | 0.0 | 53.1 |
| 2nd order | 15.0 | 0.0 | 64.3 | 37.1 | 73.9 | 0.0 | 0.0 | 68.3 | 55.4 |
| 3rd order | 20.0 | 10.0 | 77.1 | 45.8 | 71.7 | 0.0 | 0.0 | 100.0 | 57.8 |
| 4th order | 62.6 | 20.0 | 78.2 | 55.9 | 69.4 | 0.0 | 0.0 | 96.7 | 100.0 |
| 5th order | 70.4 | 20.0 | 89.3 | 63.1 | 77.7 | 20.0 | 20.0 | 100.0 | 100.0 |
| 6th order | 86.9 | 20.0 | 89.7 | 68.2 | 83.4 | 20.0 | 20.0 | 100.0 | 99.2 |
| 7th order | 87.3 | 20.0 | 98.0 | 64.6 | 88.1 | 20.0 | 20.0 | 100.0 | 99.9 |
| 8th order | 83.3 | 20.0 | 98.0 | 73.7 | 96.0 | 10.0 | 20.0 | 100.0 | 68.3 |
| group D amino acid composition | | | | | | | | | |
|  | cyt | end | ext | mit | nuc | per | pla | voc | chl |
| 1st order | 0.0 | 15.0 | 79.2 | 39.1 | 77.0 | 0.0 | 0.0 | 10.0 | 53.5 |
| 2nd order | 17.5 | 0.0 | 71.7 | 42.5 | 74.5 | 0.0 | 0.0 | 79.2 | 54.8 |
| 3rd order | 27.0 | 10.0 | 83.2 | 41.8 | 70.5 | 0.0 | 0.0 | 100.0 | 57.5 |
| 4th order | 48.6 | 20.0 | 91.0 | 59.7 | 72.1 | 0.0 | 20.0 | 100.0 | 100.0 |
| 5th order | 73.8 | 20.0 | 90.5 | 60.9 | 75.9 | 20.0 | 20.0 | 100.0 | 97.0 |
| 6th order | 84.8 | 20.0 | 97.5 | 67.1 | 83.7 | 20.0 | 20.0 | 100.0 | 99.6 |
| 7th order | 84.6 | 20.0 | 97.5 | 64.7 | 92.4 | 20.0 | 20.0 | 100.0 | 99.9 |
| 8th order | 86.0 | 20.0 | 96.7 | 78.4 | 93.7 | 10.0 | 20.0 | 100.0 | 67.2 |
| natural amino acid composition | | | | | | | | | |
|  | cyt | end | ext | mit | nuc | per | pla | voc | chl |
| one gap | 39.1 | 20.0 | 68.7 | 55.0 | 70.9 | 0.0 | 0.0 | 73.3 | 100.0 |
| two gap | 44.5 | 30.0 | 89.7 | 52.6 | 69.3 | 0.0 | 0.0 | 70.0 | 100.0 |
| three gap | 26.8 | 35.0 | 75.1 | 56.5 | 69.2 | 0.0 | 0.0 | 75.0 | 100.0 |
| four gap | 30.1 | 20.0 | 63.5 | 51.9 | 69.7 | 0.0 | 0.0 | 60.0 | 100.0 |
| five gap | 34.3 | 20.0 | 76.0 | 50.5 | 71.1 | 0.0 | 0.0 | 76.7 | 100.0 |
| six gap | 19.8 | 35.0 | 78.0 | 52.7 | 70.7 | 0.0 | 0.0 | 70.8 | 100.0 |
| group C amino acid composition | | | | | | | | | |
|  | cyt | end | ext | mit | nuc | per | pla | voc | chl |
| one gap | 10.0 | 0.0 | 50.7 | 36.1 | 70.9 | 0.0 | 0.0 | 10.0 | 53.0 |
| two gap | 0.0 | 0.0 | 79.2 | 31.5 | 70.1 | 0.0 | 0.0 | 0.0 | 53.9 |
| three gap | 0.0 | 10.0 | 53.2 | 32.5 | 71.8 | 0.0 | 0.0 | 0.0 | 54.1 |
| four gap | 0.0 | 0.0 | 58.3 | 37.3 | 71.2 | 0.0 | 0.0 | 0.0 | 53.5 |
| five gap | 0.0 | 0.0 | 80.0 | 33.2 | 69.8 | 0.0 | 0.0 | 0.0 | 52.3 |
| six gap | 0.0 | 0.0 | 80.0 | 40.3 | 65.4 | 0.0 | 0.0 | 0.0 | 54.3 |
| group D amino acid composition | | | | | | | | | |
|  | cyt | end | ext | mit | nuc | per | pla | voc | chl |
| one gap | 13.3 | 0.0 | 66.8 | 40.4 | 71.3 | 0.0 | 0.0 | 50.0 | 54.4 |
| two gap | 5.0 | 0.0 | 82.0 | 37.9 | 70.2 | 0.0 | 0.0 | 0.0 | 54.1 |
| three gap | 16.7 | 20.0 | 75.5 | 32.3 | 66.6 | 0.0 | 0.0 | 0.0 | 54.0 |
| four gap | 0.0 | 10.0 | 68.5 | 36.4 | 69.3 | 0.0 | 0.0 | 0.0 | 53.9 |
| five gap | 0.0 | 0.0 | 80.0 | 33.6 | 66.8 | 0.0 | 0.0 | 30.0 | 52.5 |
| six gap | 5.0 | 0.0 | 79.2 | 49.5 | 63.9 | 0.0 | 0.0 | 30.0 | 54.3 |
|  | | | | | | | | | |
| aaindex | 34.4 | 30.0 | 73.8 | 58.4 | 74.0 | 0.0 | 0.0 | 43.3 | 99.4 |

^1^ Abbreviations: chl, chloroplast; cyt, cytosol; end, endoplasmatic reticulum; ext, extracellular; mit, mitochondrion; nuc, nucleus; per, peroxisome; pla, plasma membrane; vac, vacuole; sn, sensitivity; ppv, positive predictive value

^2^ Bold numbers indicate the best performance for each class
