# Supplementary material for: TESTLoc: protein subcellular localization prediction from EST data
Source: BMC Bioinformatics. 2010 Nov 15;11:563. doi: 10.1186/1471-2105-11-563 (PMC3000424; doi:10.1186/1471-2105-11-563)
Supplement: Additional file 6 — The independent evaluation of SVM predictors based on different representations of amino acid composition, measured with sensitivity and positive predictive value. [file 1471-2105-11-563-S6.DOC]

**Additional file 6**. The independent evaluation of SVM predictors based on different representations of amino acid composition. A. sensitivity, B. positive predictive value. For most classes, the best result was obtained with the 4^th^ order amino acid composition (the frequency of tetra-peptides). Amino acid group-C and group-D composition yielded similar results (see Additional file 5).
