# Supplementary material for: TESTLoc: protein subcellular localization prediction from EST data
Source: BMC Bioinformatics. 2010 Nov 15;11:563. doi: 10.1186/1471-2105-11-563 (PMC3000424; doi:10.1186/1471-2105-11-563)
Supplement: Additional file 7 — Accuracy of ESTScan for the prediction of start/stop positions of coding regions in EST sequences. [file 1471-2105-11-563-S7.DOC]

**Additional file 7**. Accuracy of ESTScan for the prediction of start/stop positions of coding regions in EST sequences. The *Arabidopsis* dataset was used (see Methods). 1~10, etc, range of deviation from exact start/stop codon, counted in codons.
