# Supplementary material for: TESTLoc: protein subcellular localization prediction from EST data
Source: BMC Bioinformatics. 2010 Nov 15;11:563. doi: 10.1186/1471-2105-11-563 (PMC3000424; doi:10.1186/1471-2105-11-563)
Supplement: Additional file 8 — Comparison of prediction performance of available tools and TESTLoc on full-length plant protein sequences. [file 1471-2105-11-563-S8.DOC]

**Additional file 8.**Prediction performance of available tools and TESTLoc on full-length plant protein sequences^1^

| Predictors | chl^2^ | | cyt | | end | | ext | | mit | | nuc | | per | | pla | | vac | |
| --- | --- | --- | --- | --- | --- | --- | --- | --- | --- | --- | --- | --- | --- | --- | --- | --- | --- | --- |
|  | SN | PPV | SN | PPV | SN | PPV | SN | PPV | SN | PPV | SN | PPV | SN | PPV | SN | PPV | SN | PPV |
| TargetP | 39 | 83 |  |  |  |  | **100** | 31 | 58 | 40 |  |  |  |  |  |  |  |  |
| Pprowler | 36 | **90** |  |  |  |  | **100** | 29 | 74 | 39 |  |  | **38** | **100** |  |  |  |  |
| Bacello | 81 | 80 | **71** | 73 |  |  | 61 | 30 | 41 | **87** | **88** | 59 |  |  |  |  |  |  |
| WolfPSORT | 51 | 75 | 66 | 25 | 25 | 50 | 57 | 68 | 33 | 53 | 80 | 64 | 13 | 50 | **44** | 41 | 31 | 55 |
| TESTLoc^3^ | **92** | 78 | 62 | **100** | **75** | **100** | 96 | **100** | **80** | 65 | 74 | **99** | 0 | 0 | 13 | **100** | **74** | **100** |

^1^ Numbers in %. Bold numbers indicate the best performance for each class.

^2^Abbreviations: chl, chloroplast; cyt, cytosol; end, endoplasmatic reticulum; ext, extracellular; mit, mitochondrion; nuc, nucleus; per, peroxisome; pla, plasma membrane; vac, vacuole; SN, sensitivity; PPV, positive predictive value

^3^ The sequence feature used in TESTLoc is the integration of predictions from the three top-performing features: 4^th^ order amino acid composition, 6^th^ order group-C amino acid composition, and 7^th^ order group-D amino acid composition
